# A composite genome approach to identify phylogenetically informative data from next-generation sequencing

**DOI:** 10.1186/s12859-015-0632-y

**Published:** 2015-06-11

**Authors:** Rachel S. Schwartz, Kelly M. Harkins, Anne C. Stone, Reed A. Cartwright

**Affiliations:** 1 The Biodesign Institute, Arizona State University, Tempe, AZ USA; 2 School of Human Evolution and Social Change, Arizona State University, Tempe, AZ USA; 3 Department of Anthropology, University of California – Santa Cruz, Santa Cruz, CA USA; 4 School of Life Sciences, Arizona State University, Tempe, AZ USA

**Keywords:** Phylogenetics, Next-generation sequencing, Apes, Mammals

## Abstract

**Background:**

Improvements in sequencing technology now allow easy acquisition of large datasets; however, analyzing these data for phylogenetics can be challenging. We have developed a novel method to rapidly obtain homologous genomic data for phylogenetics directly from next-generation sequencing reads without the use of a reference genome. This software, called SISRS, avoids the time consuming steps of *de novo* whole genome assembly, multiple genome alignment, and annotation.

**Results:**

For simulations SISRS is able to identify large numbers of loci containing variable sites with phylogenetic signal. For genomic data from apes, SISRS identified thousands of variable sites, from which we produced an accurate phylogeny. Finally, we used SISRS to identify phylogenetic markers that we used to estimate the phylogeny of placental mammals. We recovered eight phylogenies that resolved the basal relationships among mammals using datasets with different levels of missing data. The three alternate resolutions of the basal relationships are consistent with the major hypotheses for the relationships among mammals, all of which have been supported previously by different molecular datasets.

**Conclusions:**

SISRS has the potential to transform phylogenetic research. This method eliminates the need for expensive marker development in many studies by using whole genome shotgun sequence data directly. SISRS is open source and freely available at https://github.com/rachelss/SISRS/releases.

**Electronic supplementary material:**

The online version of this article (doi:10.1186/s12859-015-0632-y) contains supplementary material, which is available to authorized users.

## Background

Until recently, phylogenetic studies relied on tens of loci (at most) from the genome to determine evolutionary relationships [1, 2]. However, these datasets often had insufficient information to provide strong support for all the relationships of interest [3]. Recent improvements in sequencing technology have enabled phylogenetic studies to use larger datasets in an attempt to resolve previously undetermined or controversial evolutionary relationships, but this area of research is still in its infancy [4–11].

There are currently several approaches to producing large datasets for phylogenetics. In the first approach, whole genomes are sequenced and assembled; genomes are then compared to identify homologous regions for phylogenetics [11]. The drawback of this approach is the time required to construct quality assemblies and identify homologous regions, either by annotating the genome or using genome comparison tools. Furthermore, because distantly related taxa may not be easily comparable, phylogenetic analyses using whole-genome comparisons have focused on closely related species for which alignments are possible [11, 12].

In a second approach, shotgun sequence data are aligned to a reference genome. This method assumes a reference genome, which is not always available. As with whole genome comparisons, the *de novo* assembly of a high-quality reference genome requires high-coverage data and significant time. However, even given a reference genome, homologous loci may not be recoverable for species distantly related to the reference [13].

Other approaches involve sequencing a subset of the genome. One such approach screens existing datasets for variation in the taxa of interest [14–16]. In another, regions that are conserved across taxa are identified from whole-genome alignments; both the conserved elements and regions adjacent to them may contain phylogenetic information [6, 8, 9, 17]. In a third, a consistent subsample of the genome may be sequenced [18]. However, the drawback of these approaches is that new phylogenetic markers must be developed for each research study; significant time is often required for marker development and these data have limited potential for reuse. Additionally, a consistent, phylogenetically informative subsample of the genome may be difficult to obtain at deep taxonomic levels.

Here we describe a novel computational tool, SISRS (pronounced “scissors”), to identify informative data for phylogenetic studies directly from shotgun sequencing of whole genomes. SISRS, which stands for Site Identification from Short Read Sequences, requires neither a reference genome nor *a priori* knowledge of potentially informative loci. Our software circumvents the difficulties in identifying homologous loci from whole-genome alignments when rearrangements have occurred because the conserved regions are not required to share identifiable synteny across taxa. SISRS also takes advantage of the raw data to avoid erroneously called genotypes in previously assembled genomes due to sequencing error or copy number variable regions (CNVs).

**Fig. 1:**
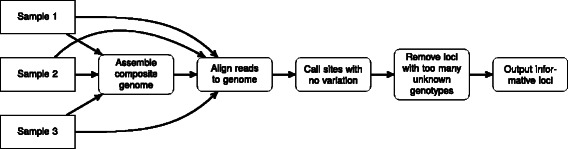
SISRS framework to identify phylogenetically informative data

SISRS identifies phylogenetically informative regions via a novel protocol (Fig. 1). (1) SISRS assembles a “composite genome” from shotgun sequencing reads for all taxa. (2) The composite genome is used as a reference to align the sequencing data for each sample. (3) The sequence for each sample is identified via a strict consensus (i.e. sites that are variable are called as unknown). (4) SISRS removes loci that have too few sites with callable genotypes (as specified by the user). In this way, SISRS identifies sites across entire genomes that are phylogenetically informative and reduces errors due to biological and experimental error.

We demonstrate that SISRS provides high quality phylogenetic datasets across a range of simulated and empirical data. First, the data output by SISRS for simulated shotgun reads was congruent with the starting phylogeny at all depths in the tree. Second, using previously sequenced shotgun data for seven primate taxa, we were able to rapidly identify homologous data using SISRS and estimate the known phylogeny accurately. Third, we used available data to estimate the phylogeny of mammals, the root of which has remained controversial. Using SISRS, phylogenies can be produced from next-generation sequencing reads in a matter of days. For example, identifying hundreds of thousands of variable sites for phylogenetics from over 100 Gb of raw mammalian transcriptome reads took less than four days.

## Results

### Recovery of phylogenetically informative sites

**Fig. 2:**
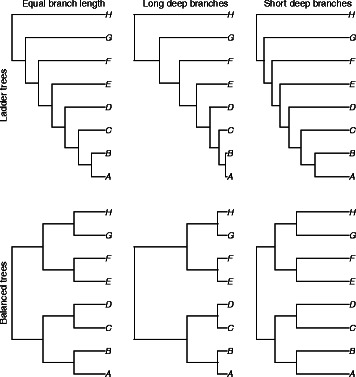
Simulation trees used to test the SISRS pipeline. All branches for the leftmost trees were initially set to 0.01 substitutions per site. Branch lengths for the center trees (long-deep) were obtained by scaling the leftmost (equal-branch-length) tree to a height of 1, then raising each node height to a power of two, and multiplying by the original tree height [31]. The rightmost (short-deep) tree was obtained similarly, but using a power of one-half. For each of the trees, branch lengths were scaled by multiplying by values from one to six

**Fig. 3:**
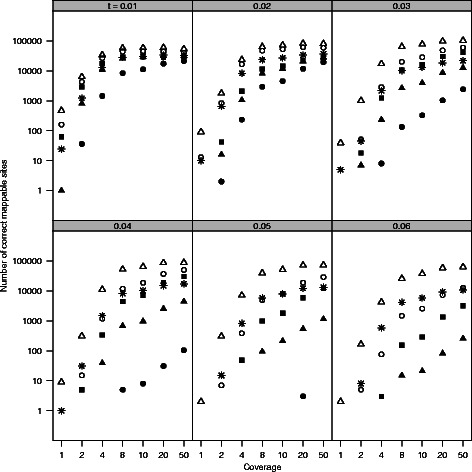
SISRS produced substantial amounts of informative data for phylogenies of different shapes and evolutionary distances. The number of true variable sites identified from simulated data is shown for each of the six simulation trees (symbols; Fig. 2) for increasing numbers of substitutions between species (panels; increasing values of t), and increasing levels of coverage (x axis). Symbols are ∘ for balanced trees; △ for balanced trees with long deep branches; ■ for balanced trees with short deep branches; $\blacktriangle $ for ladder trees; ∗ for ladder trees with long deep branches; ∙ for ladder trees with short deep branches. These sites were identified from 1 million base pair genomes; thus, larger genomes are expected to produce more sites, particularly as long as a fraction of those genomes are reasonably conserved

**Fig. 4:**
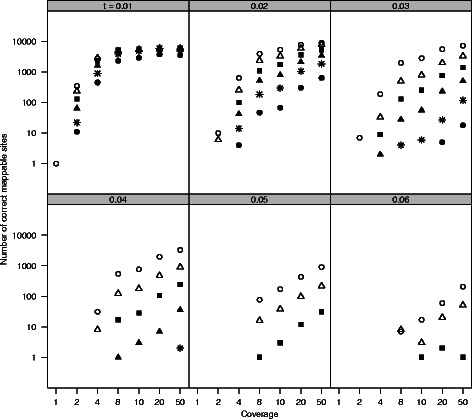
SISRS identified fewer informative sites for deeper nodes in the tree; however, some sites identified each split in all cases except for extremely diverged species with low-coverage data. Results are separated by branch length (panels), coverage (x axis), and tree depth (symbols). Only results for the equal-branch-length ladder tree are shown; results for the other ladder trees were similar (Additional file 1: Figure S1). The number of sites supporting the node A+B are denoted as ∘; △ denotes sites supporting A+B+C; ■ denotes A+B+C+D; $\blacktriangle $ denotes A+B+C+D+E; ∗ denotes A+B+C+D+E+F; ∙ denotes A+B+C+D+E+F+G. Note that these symbols refer to different results that the previous figure. Fewer sites were recovered for the tree with short deep branches, compared to the equal-branch-length tree, while more sites were recovered for the tree with long branches

**Fig. 5:**
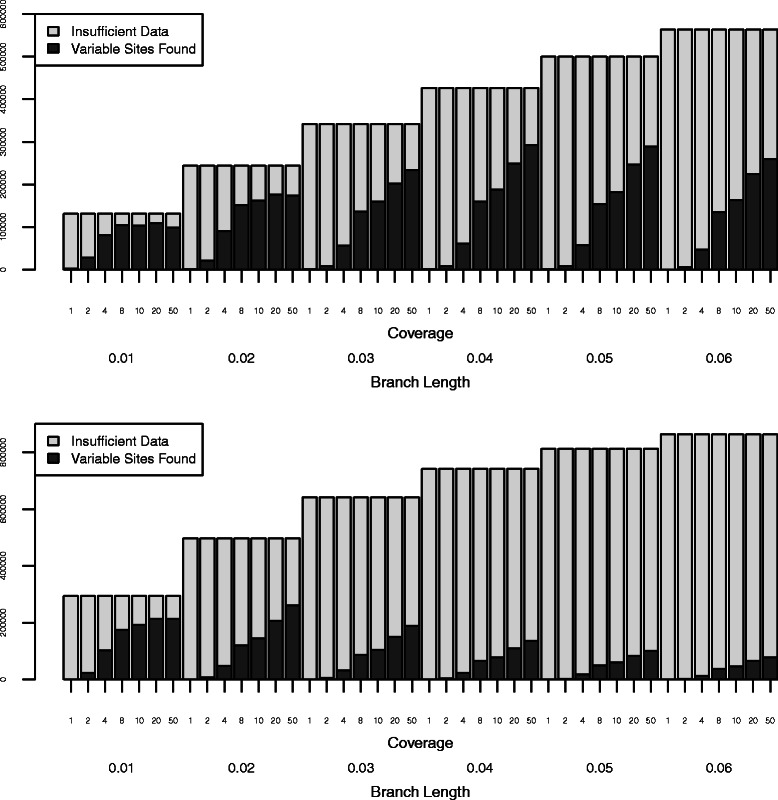
SISRS failed to identify some variable sites in simulated data due to low coverage at those sites or lack of coverage by the composite genome. Top: Results for the balanced tree with equal-length branches. Bottom: Results for the ladder tree with equal-length branches. The results for the remaining trees are nearly identical

To determine how well our approach identified phylogenetically informative data, we simulated genomes on 36 phylogenies. These phylogenies included two topologies (ladder shaped and balanced) with three variants each: equal-length branches, longer deep branches, and shorter deep branches (Fig. 2). Each tree was rescaled by multiplying the branch length by a scaler of one to six. We then simulated NGS data on these genomes and examined how well we were able to recover variable sites. For all simulation trees, the number of potentially informative sites identified using SISRS increased with increased coverage (Fig. 3). As the distance between taxa (i.e. branch length) increased for a given tree, the number of output sites decreased (Fig. 3). Of these sites, a plurality allowed the accurate identification of the shallowest nodes within a tree, with a decreasing number allowing the identification of deeper nodes (Fig. 4; Additional file 1: Figure S1). Insufficient coverage in the simulations, lack of coverage by the composite genome, and insufficient coverage following mapping of the reads to the composite genome reference contributed to a failure to recover all variable sites (Fig. 5). Potential false positives due to read simulation error were removed by SISRS as part of calling by strict consensus; however, some new false positives were introduced, likely due to erroneous mapping of reads to the composite genome. These false positives represented less than 1 % of the sites found for any tree; thus, they have little effect on phylogenetic inference.

### Ape tree is recovered

**Fig. 6:**
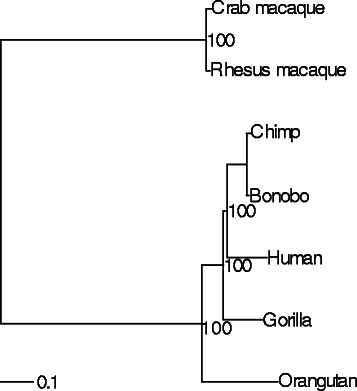
The phylogeny generated with RAxML 8.0 for apes based on an alignment of variable sites produced by SISRS with no more than one unknown genotype per site. The phylogeny is based on 1000 bootstrap datasets (bootstrap percentages are shown at nodes)

We also tested the utility of SISRS using NGS data from apes, for which the phylogeny is well established. This analysis was conducted using 14 cores on a FreeBSD 10.0 server; the total time to produce an alignment from raw reads was 36 hours. The maximum amount of memory required during the composite genome assembly was 1.5 Gb. We identified 148,639 variable sites that contained observations in at least five samples. The ML estimate of the phylogeny with 1000 bootstraps was fully concordant with the known phylogeny of apes with all nodes supported at 100 % (Fig. 6).

### Mammal phylogeny

We further tested the utility of SISRS using NGS data from placental mammals, for which the phylogeny is controversial. This analysis was conducted using 40 cores; the total time to produce the composite genome was less than an hour; the remaining alignment, base calling, and site identification steps required an additional 87 hours. 10 Gb of memory was required during the composite genome assembly. The maximum amount of memory required to process the data mapped to the composite genome was 45 Gb; however, because data processing was conducted across multiple cores to increase the speed of the analysis, the total amount of memory used at one time by SISRS was over 300 Gb. Thus, this analysis could be conducted using fewer resources over more time.

**Fig. 7:**
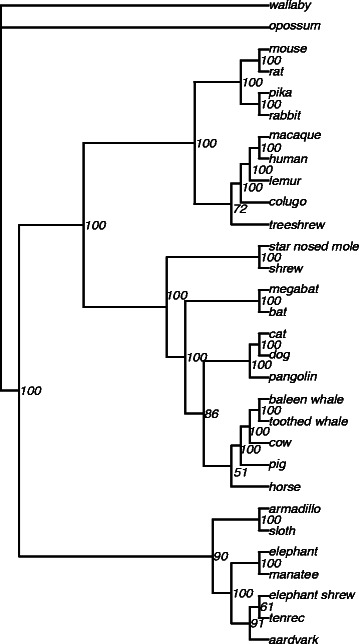
The phylogeny generated with RAxML 8.0 for placental mammals based on an alignment of variable sites produced by SISRS with no more than ten unknown genotypes per site. The phylogeny is a majority rule consensus based on 1000 bootstrap datasets

We produced 15 alignments, each allowing a set number of unknown genotypes at each site (i.e. alignment 1 has no more than one species with an unknown genotype). The number of sites in the alignments ranged from 21 to over 1.5 million. Analyzed in a ML framework, the first four alignments produced phylogenies with multiple polytomies due to limited data; they are not described further. The remaining alignments produced phylogenies that reflect previous conflicting estimates of the relationships among mammals. For example, in regards to the basal relationships alignments 5, 10, 11, and 12 supported Xenarthra+Afrotheria (Atlantogenata) as a clade sister to all other mammals [19–21], alignment 6 supported Xenarthra as a separate clade [22], alignments 13, 14, and 15 supported Afrotheria as a separate clade [9, 23], and the remaining alignments did not resolve these relationships. Similarly, the relationship of the treeshrew to other mammals was difficult to resolve: for some alignments this species formed a clade with rodents, while for others it formed a clade with primates. The majority rule consensus phylogeny generated from alignment 10 (i.e. no more than 10 species had an unknown genotype at each site) is shown in Fig. 7; additional phylogenies are shown in Additional file 2: Figure S2.

## Discussion

### Data produced by SISRS

For simulations, SISRS was able to recover large numbers of variable sites, unless branch lengths were unreasonably long or coverage was low. Based on these results, coverage should average 5–10x for optimal marker identification; however, low coverage sequencing will also identify useful data. Most genomes are much larger than the one million bases in our simulations; far more sites will be identified for larger genomes, making the use of low coverage data feasible.

Identifying homologous variable sites was more challenging for trees with long branches (i.e. large evolutionary distances as measured by number of substitutions per site between pairs of taxa). Large numbers of substitutions at a region in the genome will result in difficulties assembling this region as a single locus to which data from all species can be aligned. Instead, we expect that data from the most similar species (e.g. A–B in the pectinate trees) will be assembled into a consensus contig; the distance between these species for the longest equal-branch-length pectinate tree is 0.12 substitutions per site. However, the distance from C to any other species is at least 0.24 substitutions per site (on average), and distances are longer for the remaining species. Thus, the contigs are expected to be assembled jointly from A/B, and independently from data for each of the remaining species (if assembly is possible given low-coverage subsampling of reads). Consequently, data from each species for a region of the genome will either align to different contigs (assembled independently from different species) or fail to align entirely. This process results in difficulty identifying homologous data using SISRS when branch lengths are long.

However, this result is less problematic for empirical data. In real genomes, unlike our simulations, loci evolve at different rates; thus, there will likely always be some loci for which the branch length (in substitutions per site) between taxa is very small. These sites will be identified by SISRS.

As expected, deeper nodes were more difficult to recover, likely because the synapomorphies between the two clades may be overwritten by new substitutions. However, the sites that were identified are informative about these relationships. Overall, the concordance of the data with the simulation trees demonstrates that the SISRS approach produces extensive phylogenetically informative data for deep and shallow evolutionary time scales.

### Empirical results

Using available NGS data we were able to recover the phylogeny of apes quickly and accurately. Similarly, using available NGS data we recovered a mammal phylogeny reflecting previous conflicting estimates of the relationships among mammals [12, 21, 23, 24]. These conflicting estimates, particularly in regards to the basal relationships and the position of the treeshrew, were all found using “traditional” phylogenetic methods.

### Advantages of a composite genome

Generating a composite genome has multiple advantages over aligning data to a reference genome to identify potential phylogenetically informative sites. First, an assembled reference genome similar to the taxa of interest is not always available. Second, assembling a reference genome requires high coverage data from at least one species and is time consuming; assembling the whole genome is necessary because it is impossible to determine *a priori* regions of the genome that may be phylogenetically informative. In contrast, SISRS does not require high levels of coverage or a time consuming assembly. A composite reference genome containing phylogenetically-informative homologous regions can be assembled in a few hours. Furthermore, when taxa are highly diverged, data may align poorly to a single reference genome. In contrast, a composite genome contains data from all taxa, allowing better alignment of all data across the phylogeny. Because each species is subsampled for the assembly, unique regions will be limited in the final assembly, while maintaining an optimal assembly for conserved regions. Within each conserved region, the composite genome contains sites with the most common base, making it more likely that data from all taxa will align to this region.

### Time required

The time to run SISRS is highly variable, depending on the number of processors available, the number of samples sequenced, and the amount of data sequenced per sample. Given large numbers of processors (e.g. a cluster of >30 nodes), SISRS makes phylogenetic analysis from shotgun data possible within a few days. Even given the limitations of a desktop computer, it is possible to produce many phylogenies within a couple of weeks. Unlike other phylogenetic methods, SISRS entirely avoids the weeks required for marker development or sample processing; preparation for sequencing and the sequencing time itself are required for all phylogenomic approaches. Alternatively, as with the analyses conducted here, all time required for sequencing and preparation was avoided entirely by using data made available from other research projects.

### Data analysis

The approaches we have used to analyze our data are not designed for large datasets of variable sites, although our results suggest that with the exception of short deep internodes the recovery of the phylogeny is quite good. Ideal methods would accommodate differences among gene trees to correctly estimate the species tree and model the substitution process to accurately infer substitutions. However, current methods to analyze genome-wide variable sites are limited [25]. It is important to note that these methods are in development; as we begin to use whole-genome data it is obvious that subsets of data must be used and the optimal data are likely not linked regions, but individual sites [11]. Furthermore, the availability of tools designed for limited datasets should not prevent us from developing methods to identify more comprehensive datasets.

### Future directions

Future versions of SISRS will accommodate larger genomes and output more variable sites more rapidly as a result of improved assembly of the composite genome and improved genotype calling. We will also evaluate the application of SISRS output to deep-time phylogenetics and estimation of branch lengths/divergence times among taxa.

## Conclusions

The approach introduced here has the potential to transform phylogenetic research. SISRS eliminates the need for expensive marker development in many studies by using whole genome shotgun sequence data directly. As technology improves, whole-genome sequencing will soon be affordable even for large-scale projects. By using shotgun sequence data, error in next-generation sequence data and co-alignment of paralogous genes does not affect subsequent analyses.

SISRS also promotes the reuse of data. Shotgun genomic sequences available in public databases can be used directly for phylogenetic analyses, as we have done in this study. Sequencing performed with the goal of identifying phylogenetic data using SISRS can be made available for subsequent use in other studies, including phylogenetics at any taxonomic level, or any other study utilizing genomic data. Reusing available next-generation sequencing reads can substantially reduce costs.

## Methods

### Composite reference genome

SISRS currently uses a de Bruijn-graph-based de novo assembler, Velvet [26], to construct a composite reference genome. Analysis of alternative algorithms is ongoing. The composite genome is assembled from a subset of raw NGS reads (i.e. FASTQ files) from multiple taxa. When using a subset of the data, conserved regions are likely to be assembled. Regions of the genome that are either unique to a single taxon or highly variable among taxa are less likely to be included in the composite genome. To construct the subset, SISRS uses reservoir sampling [27] from the data for each species. The size of the subsample is determined based on a user-specified genome size such that the subsample includes approximately 10x coverage of conserved regions across species. After assembling these diverse data, the resulting contigs contain composite sequences of loci that are conserved across some or all taxa. Using a subset of the raw data also significantly reduces Velvet’s memory footprint.

### Site calling for each position in the reference

To determine conserved regions and variation among species within these regions, the full sequencing data is mapped back to the composite reference genome using Bowtie 2 [28]. SISRS uses a strict consensus to call the genotype of each species for each site in the composite reference. This is a conservative approach to eliminate any sites that contain paralogous data (i.e. false positive variable sites), thus significantly reducing non-phylogenetic signal [29]. Both subsampling (described above) and genotype calling are sped up using GNU Parallel [30]. After genotype calling, SISRS produces a dataset containing sites that have information for most or all taxa (as specified by the user). To reduce the final dataset, SISRS can produce a final alignment containing only sites that are variable among taxa; this type of data is used in all analyses described below.

### Simulations to test methodology

To determine how well our approach identified phylogenetically informative sites, we simulated 252 datasets of next-generation sequencing reads with different levels of sequencing coverage on multiple phylogenies. We used six eight-taxon phylogenies to simulate genomes. Three of the trees were pectinate (ladder shaped); the other three were balanced. For each tree shape (pectinate or balanced), three trees were generated with (1) equal internode branch lengths, (2) decreasing internode branch lengths from root to tip, and (3) increasing internode branch lengths from root to tip (Fig. 2). Relative branch lengths for the long-deep-branch length and short-deep-branch length trees were generated by scaling the equal-branch-length tree to a height of 1, then raising each node height to a power of one-half or two [31]. We produced a total of 36 trees with increasing levels of divergence among species by multiplying each branch length by values from 0.01 to 0.06. We simulated genomes of one million nucleotides on each of these trees using the Jukes-Cantor model with Dawg 2.0 [32, 33]. Illumina-like NGS data were simulated using the software ART (version BananaPancakes-04-02-2013) with included MiSeq error model [34]. Each simulation had either 1, 2, 4, 8, 10, 20, or 50x coverage; reads were 100 bp and paired-end. For each data set we recorded the total number of variable sites simulated, the total number of variable sites output by SISRS, and the number of these sites that could be mapped. We expected some variable sites not to be recovered by SISRS due to insufficient data in the simulations, lack of coverage by the composite genome, and poor read mapping. To determine the value of the data output by SISRS we also counted the number of sites that were concordant with the true tree as a function of depth.

### Empirical data test

We further tested our approach using data from apes, including human (*Homo sapiens*), chimpanzee (*Pan troglodytes*), bonobo (*P. paniscus*), gorilla (*Gorilla gorilla* and *G. beringei*), and orangutan (*Pongo pygmaeus* and *P. abelii*). The crab-eating macaque (*Macaca fascicularis*) and rhesus macaque (*M. mulatta*) were used to root the tree. These primates were chosen to test the efficiency and effectiveness of this method on empirical data due to their well-established phylogeny [35].

Raw Illumina paired-end sequence data were obtained from the European Nucleotide Archive and the 1000 Genomes Project [36] (Additional file 3: Table S1). We combined data from two individuals for each species. To reduce the size of the dataset being analyzed, we aligned the data to the human genome (build 37) using Bowtie 2 as in SISRS. We extracted only the data that aligned to human chromosome 21. These reads were then placed in FASTQ files as new paired-end datasets, as would be generated directly from a sequencing run. Potentially informative sites were obtained using SISRS. The genome size specified for the composite genome subsampling procedure was 48 million, approximately the size of human chromosome 21.

Due to the size of the dataset, we treated the data output by SISRS as a single concatenated locus [11] and analyzed the data in a maximum likelihood (ML) framework with 1000 bootstraps implemented in RAxML-HPC2 8.0.3 [37]. The GTRGAMMA model was used; the omission of invariable sites was accommodated with the ASC parameter, which results in calculating likelihoods assuming all sites are variable [38].

### Estimating the mammal phylogeny

We further demonstrate the value of the SISRS approach using 30 placental mammal taxa. Transcriptome and genome data were obtained as above (Additional file 3: Table S1). For most taxa we combined data from two or three individuals to increase genome coverage. Potentially informative sites were obtained using SISRS; however, the composite genome was derived exclusively from transcriptome data to reduce assembly time and memory requirements. The genome size specified for the composite genome subsampling procedure was 100 million. The dataset output by SISRS for mammals was analyzed using the same method as the ape dataset.

No ethical approval was required for any aspect of the study.

## Additional files

Additional file 1
**Figure S1.** SISRS identified fewer informative sites for deeper nodes in the tree; however, in most cases the number of sites was sufficient to resolve the tree. Results are separated by branch length (panels), coverage (x axis), and tree depth (symbols). (a) Ladder trees. Top: results for the equal-branch-length tree. Middle: results for the tree with short deep branches. Bottom: results for the tree with long deep branches. The number of sites supporting the node A+B are denoted as ∘; △ denotes sites supporting A+B+C; ■ denotes A+B+C+D; $\blacktriangle $ denotes A+B+C+D+E; ∗ denotes A+B+C+D+E+F; ∙ denotes A+B+C+D+E+F+G. (b) Balanced trees. Data are given in the same order. The number of sites supporting the node A+B, C+D, E+F, or G+H are denoted as ∘; △ denotes sites supporting A+B+C+D or E+F+G+H.

Additional file 2
**Figure S2.** Phylogenies estimated for placental mammals using data from SISRS with RAxML 8.0. The datasets were missing information for up to 5, 6, 7, 8, 9, 11, 12, 13, 14, and 15 species at each site respectively.

Additional file 3
**Table S1.** Accession numbers for data downloaded from the European Nucleotide Archive. Human data are from the 1000 genomes project [36]. Taxa for which transcriptome data was used are italicized.

## Abbreviations

ML: Maximum likelihood
NGS: Next generation sequencing
SISRS: Site identification from short read sequences

## References

[1] Giribet G, Edgecombe GD, Wheeler WC (2001). Arthropod phylogeny based on eight molecular loci and morphology. Nature.

[2] Harpke D, Meng S, Rutten T, Kerndorff H, Blattner FR (2013). Phylogeny of *Crocus* (Iridaceae) based on one chloroplast and two nuclear loci: ancient hybridization and chromosome number evolution. Mol Phylogenet Evol.

[3] Stanley EL, Bauer AM, Jackman TR, Branch WR, Le Fras N Mouton P (2011). Between a rock and a hard polytomy: rapid radiation in the rupicolous girdled lizards (Squamata: Cordylidae). Mol Phylogenet Evol.

[4] Burleigh JG, Bansal MS, Eulenstein O, Hartmann S, Wehe A, Vision TJ (2011). Genome-scale phylogenetics: inferring the plant tree of life from 18,896 gene trees. Syst Biol.

[5] Cohen E, Chor B (2012). Detecting phylogenetic signals in eukaryotic whole genome sequences. J Comput Biol.

[6] Crawford NG, Faircloth BC, McCormack JE, Brumfield RT, Winker K, Glenn TC (2012). More than 1000 ultraconserved elements provide evidence that turtles are the sister group of archosaurs. Biol Lett.

[7] Delsuc F, Brinkmann H, Philippe H (2005). Phylogenomics and the reconstruction of the tree of life. Nat Rev Genet.

[8] Faircloth BC, McCormack JE, Crawford NG, Harvey MG, Brumfield RT, Glenn TC (2012). Ultraconserved elements anchor thousands of genetic markers spanning multiple evolutionary timescales. Syst Biol.

[9] McCormack JE, Faircloth BC, Crawford NG, Gowaty PA, Brumfield RT, Glenn TC (2012). Ultraconserved elements are novel phylogenomic markers that resolve placental mammal phylogeny when combined with species-tree analysis. Genome Res.

[10] McCormack JE, Harvey MG, Faircloth BC, Crawford NG, Glenn TC, Brumfield RT (2013). A phylogeny of birds based on over 1,500 loci collected by target enrichment and high-throughput sequencing. PloS One.

[11] Yoder JB, Briskine R, Mudge J, Farmer A, Paape T, Steele K, Weiblen GD, Bharti AK, Zhou P (2013). Phylogenetic signal variation in the genomes of *Medicago* (Fabaceae). Syst Biol.

[12] Fan Y, Huang Z, Cao C, Chen C, Chen Y, Fan D, He J, Hou H, Hu L, Hu X, Jiang X, Lai R, Lang Y, Liang B, Liao S, Mu D, Ma Y, Niu Y, Sun X, Xia J, Xiao J, Xiong Z, Xu L, Yang L, Zhang Y, Zhao W, Zhao X, Zheng Y, Zhou J, Zhu Y, Zhang G, Wang J, Yao Y (2013). Genome of the chinese tree shrew. Nat Commun.

[13] Bertels F, Silander OK, Pachkov M, Rainey PB, Nimwegen van E (2014). Automated reconstruction of whole-genome phylogenies from short-sequence reads. Mol Biol Evol.

[14] O’Neill EM, Schwartz R, Bullock CT, Williams JS, Shaffer HB, Aguilar-Miguel X, Parra-Olea G, Weisrock DW (2013). Parallel tagged amplicon sequencing reveals major lineages and phylogenetic structure in the North American tiger salamander (*Ambystoma tigrinum*) species complex. Mol Ecol.

[15] Senn H, Ogden R, Cezard T, Gharbi K, Iqbal Z, Johnson E, Kamps-Hughes N, Rosell F, McEwing R (2013). Reference-free SNP discovery for the Eurasian beaver from restriction site-associated DNA paired-end data. Mol Ecol.

[16] Steele PR, Guisinger-Bellian M, Linder CR, Jansen RK (2008). Phylogenetic utility of 141 low-copy nuclear regions in taxa at different taxonomic levels in two distantly related families of rosids. Mol Phylogenet Evol.

[17] Lemmon AR, Emme SA, Lemmon EM (2012). Anchored hybrid enrichment for massively high-throughput phylogenomics. Syst Biol.

[18] Eaton DAR, Ree RH (2013). Inferring phylogeny and introgression using RADseq data: an example from flowering plants (*Pedicularis*: Orobanchaceae). Syst Biol.

[19] Meredith RW, Janečka JE, Gatesy J, Ryder OA, Fisher CA, Teeling EC, Eizirik E, Simão TL, Stadler T (2011). Impacts of the cretaceous terrestrial revolution and KPg extinction on mammal diversification. Science.

[20] Song S, Liu L, Edwards SV, Wu S (2012). Resolving conflict in eutherian mammal phylogeny using phylogenomics and the multispecies coalescent model. Proc Nat Acad Sci.

[21] Morgan CC, Foster PG, Webb AE, Pisani D, McInerney JO, O’Connell MJ (2013). Heterogeneous models place the root of the placental mammal phylogeny. Mol Biol Evol.

[22] O’Leary MA, Bloch JI, Flynn JJ, Gaudin TJ, Giallombardo A, Giannini NP, Goldberg SL, Kraatz BP, Luo Z-X, Meng J. (2013). The placental mammal ancestor and the post-K-Pg radiation of placentals. Science.

[23] Romiguier J, Ranwez V, Delsuc F, Galtier N, Douzery EJP (2013). Less is more in mammalian phylogenomics: AT-rich genes minimize tree conflicts and unravel the root of placental mammals. Mol Biol Evol.

[24] Teeling EC, Hedges SB (2013). Making the impossible possible: Rooting the tree of placental mammals. Mol Biol Evol.

[25] Bryant D, Bouckaert R, Felsenstein J, Rosenberg NA, RoyChoudhury A (2012). Inferring species trees directly from biallelic genetic markers: bypassing gene trees in a full coalescent analysis. Mol Biol Evol.

[26] Zerbino DR, Birney E (2008). Velvet: algorithms for *de novo* short read assembly using de Bruijn graphs. Genome Res.

[27] Vitter JS (1985). Random sampling with a reservoir. ACM T Math Softw.

[28] Langmead B, Salzberg SL (2012). Fast gapped-read alignment with Bowtie 2. Nat Methods.

[29] Philippe H, Brinkmann H, Lavrov D, Littlewood D, Manuel M, Worheide G, Baurain D (2011). Resolving difficult phylogenetic questions: why more sequences are not enough. PLoS Biol.

[30] Tange O (2011). GNU Parallel - the command-line power tool ;login:. USENIX Mag.

[31] Grafen A (1989). The phylogenetic regression. Philos Trans R Soc Lond B Biol Sci.

[32] Cartwright RA (2005). DNA assembly with gaps (Dawg): simulating sequence evolution. Bioinformatics.

[33] Jukes TH, Cantor CR, Munro HN, Munro HN (1969). Evolution of protein molecules. Mammalian Protein Metabolism.

[34] Huang W, Li L, Myers JR, Marth GT (2012). ART: a next-generation sequencing read simulator. Bioinformatics.

[35] Perelman P, Johnson WE, Roos C, Seuánez HN, Horvath JE, Moreira MAM, Kessing B, Pontius J, Roelke M, Rumpler Y, Schneider MPC, Silva A, O’Brien SJ, Pecon-Slattery J (2011). A molecular phylogeny of living primates. PLoS Genet.

[36] 1000 Genomes Project Consortium (2010). A map of human genome variation from population-scale sequencing. Nature.

[37] Stamatakis A (2006). RAxML-VI-HPC: maximum likelihood-based phylogenetic analyses with thousands of taxa and mixed models. Bioinformatics.

[38] Lewis PO (2001). A likelihood approach to estimating phylogeny from discrete morphological character data. Syst Biol.

