# Supplementary figures and images for: A composite genome approach to identify phylogenetically informative data from next-generation sequencing
Source: BMC Bioinformatics. 2015 Jun 11;16:193. doi: 10.1186/s12859-015-0632-y (PMC4464851; doi:10.1186/s12859-015-0632-y)

(a)

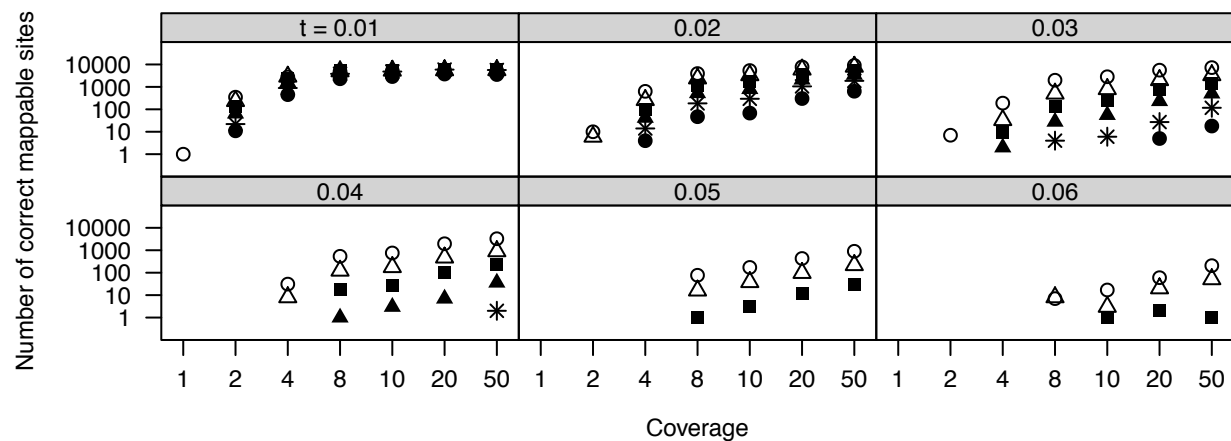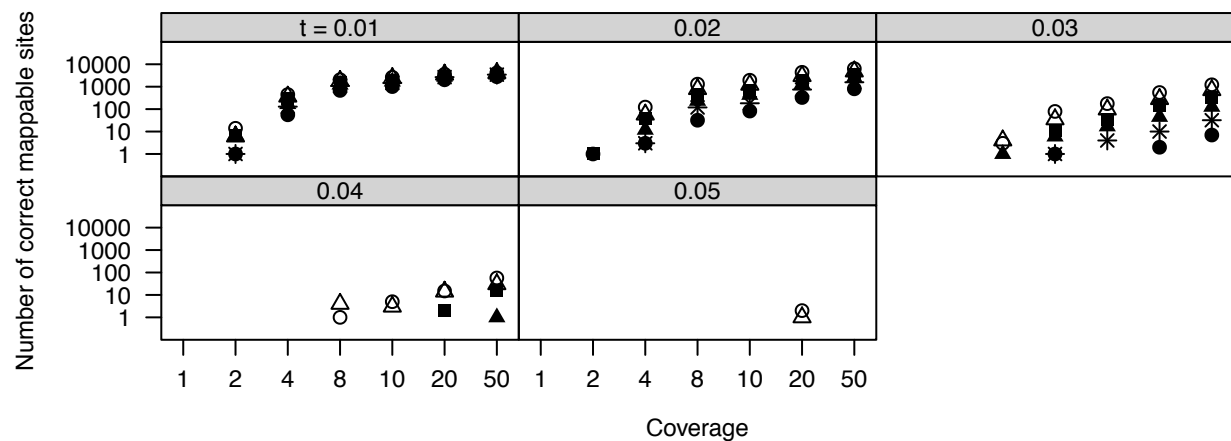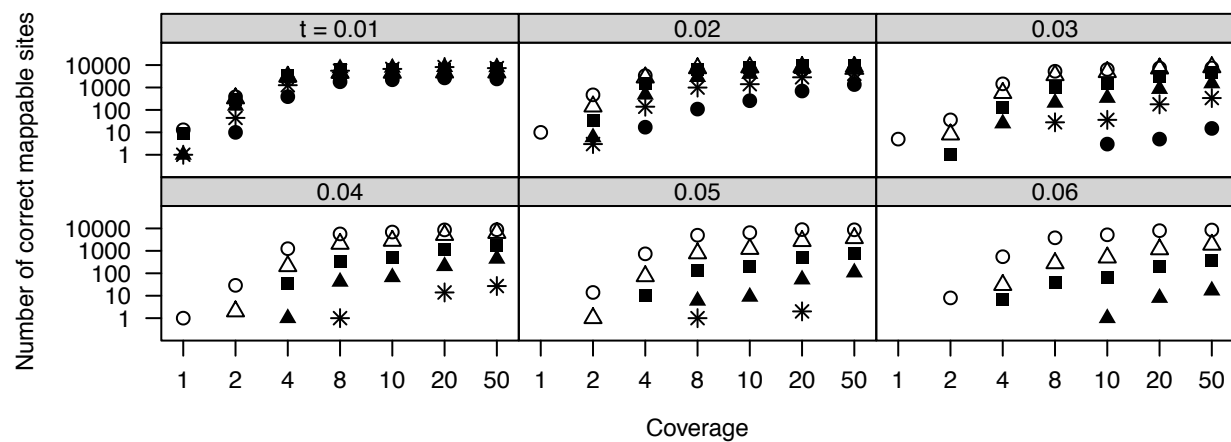

(b)

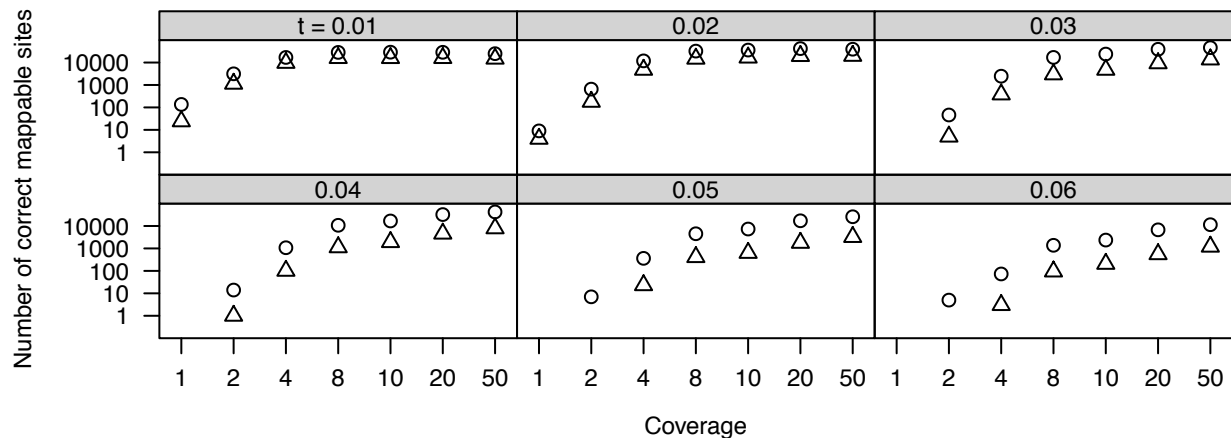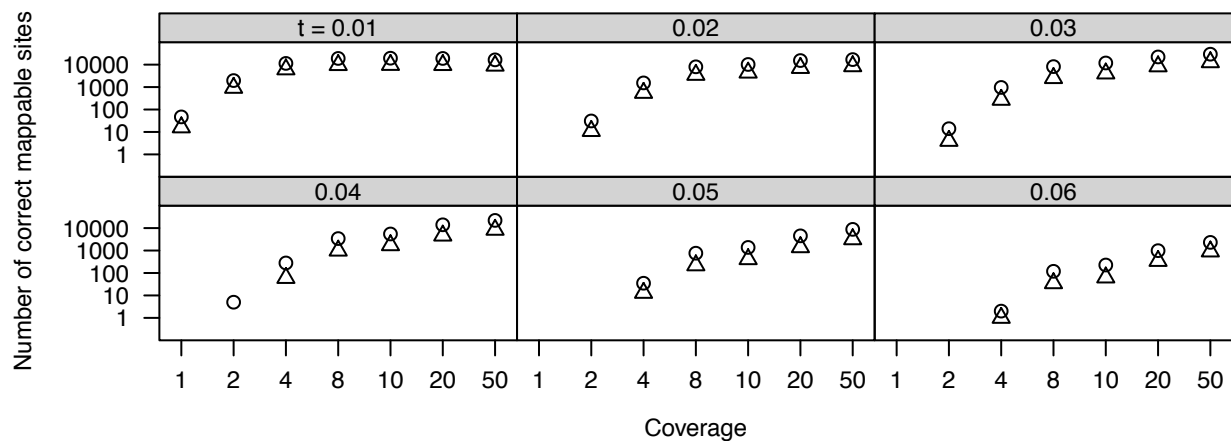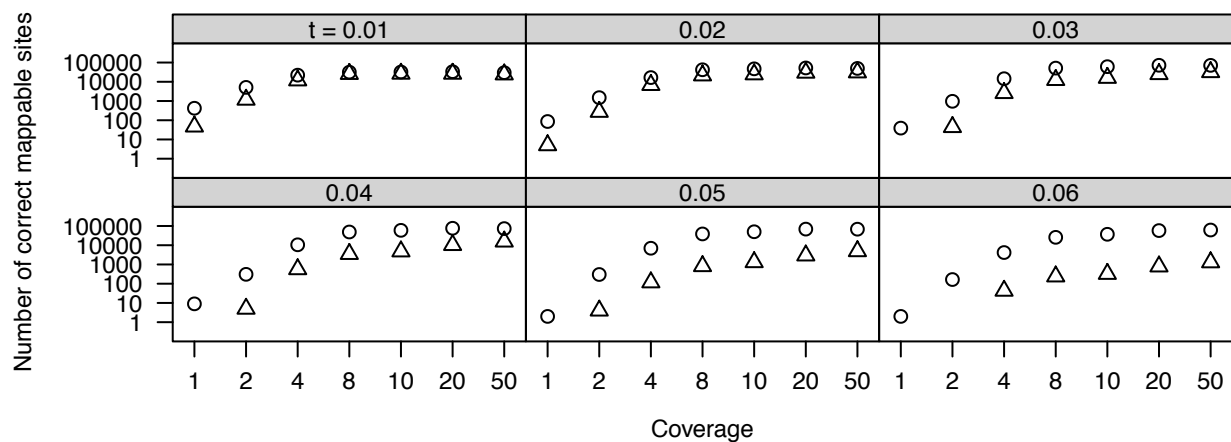

Supplement: Additional file 1 — Figure S1. SISRS identified fewer informative sites for deeper nodes in the tree; however, in most cases the number of sites was sufficient to resolve the tree. Results are separated by branch length (panels), coverage (x axis), and tree depth (symbols). (a) Ladder trees. Top: results for the equal-branch-length tree. Middle: results for the tree with short deep branches. Bottom: results for the tree with long deep branches. The number of sites supporting the node A+B are denoted as ∘; △ denotes sites supporting A+B+C; ■ denotes A+B+C+D; $\blacktriangle$▴ denotes A+B+C+D+E; ∗ denotes A+B+C+D+E+F; ∙ denotes A+B+C+D+E+F+G. (b) Balanced trees. Data are given in the same order. The number of sites supporting the node A+B, C+D, E+F, or G+H are denoted as ∘; △ denotes sites supporting A+B+C+D or E+F+G+H. [file 12859_2015_632_MOESM1_ESM.pdf]

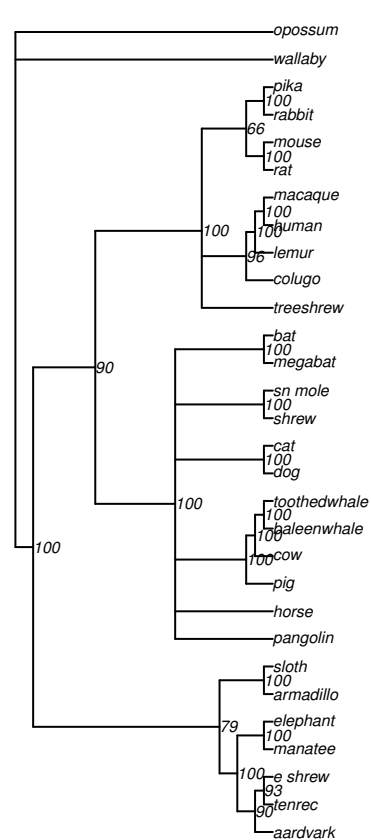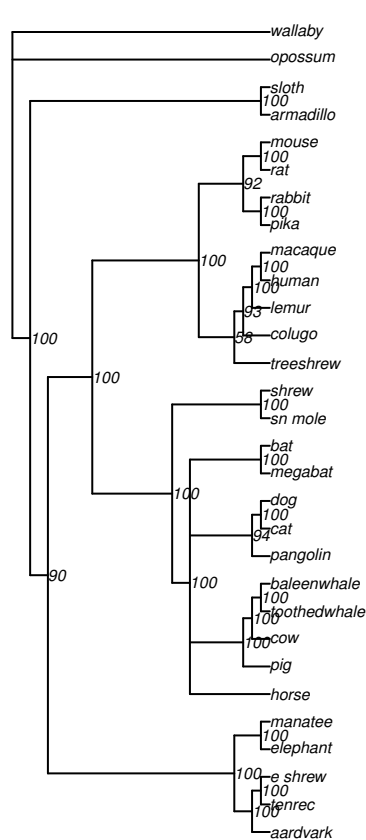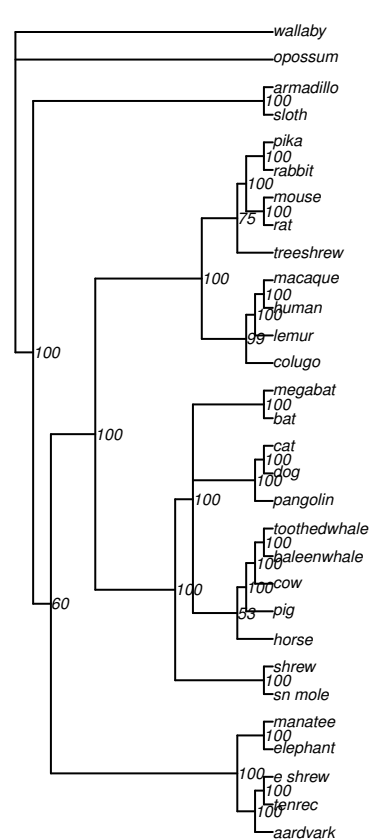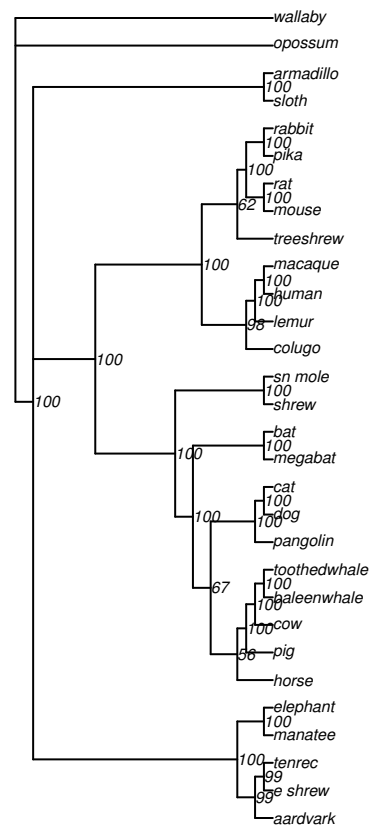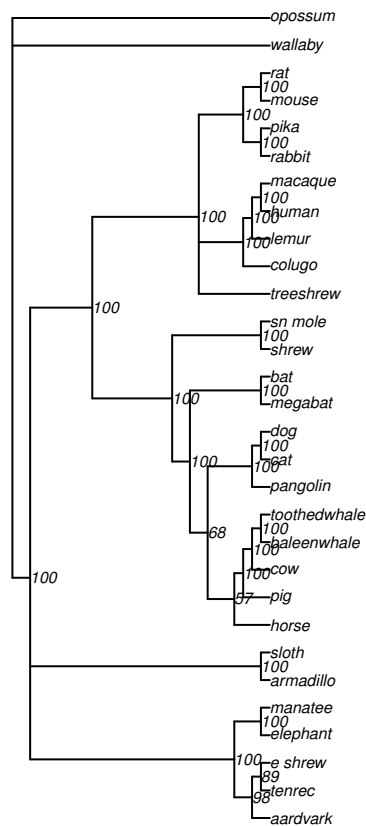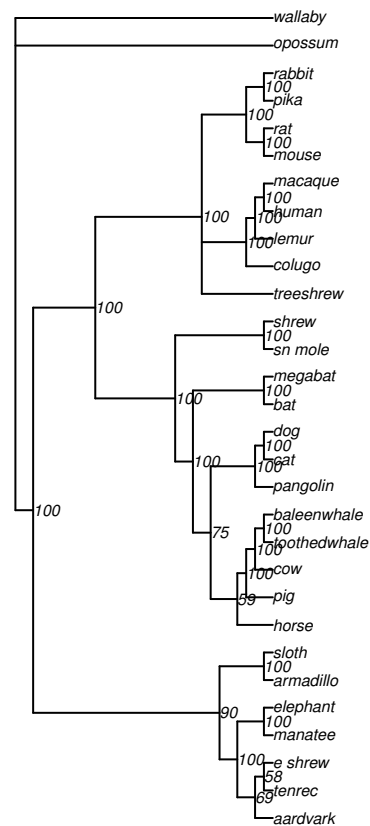

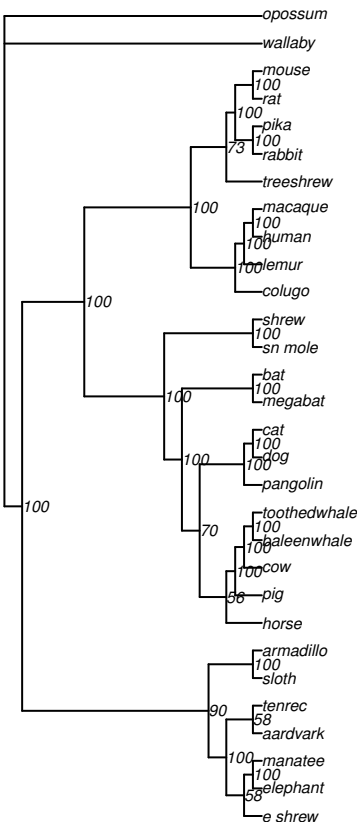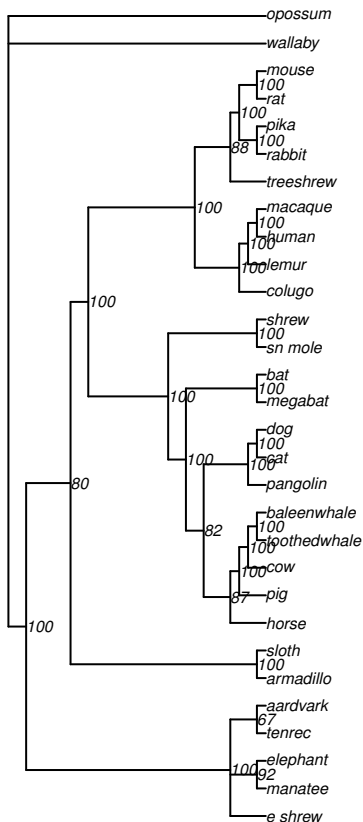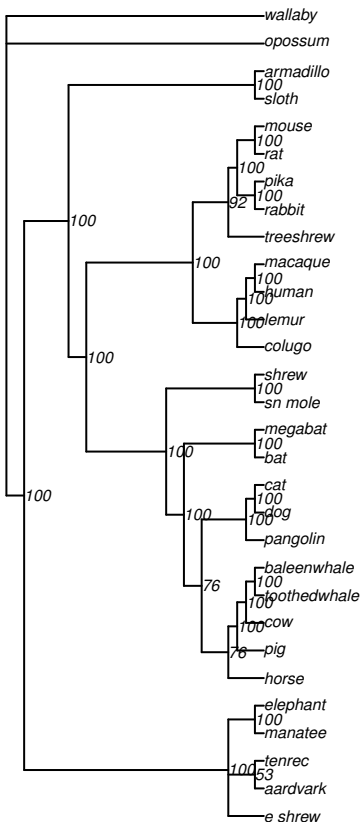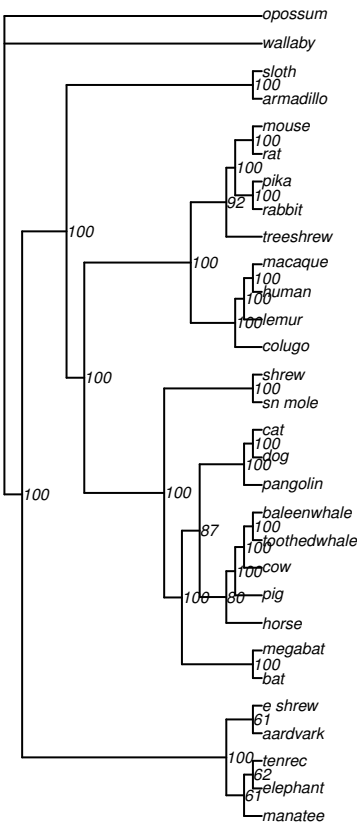

Supplement: Additional file 2 — Figure S2. Phylogenies estimated for placental mammals using data from SISRS with RAxML 8.0. The datasets were missing information for up to 5, 6, 7, 8, 9, 11, 12, 13, 14, and 15 species at each site respectively. [file 12859_2015_632_MOESM2_ESM.pdf]
