# Supplementary material for: A composite genome approach to identify phylogenetically informative data from next-generation sequencing
Source: BMC Bioinformatics. 2015 Jun 11;16:193. doi: 10.1186/s12859-015-0632-y (PMC4464851; doi:10.1186/s12859-015-0632-y)
Supplement: Additional file 3 — Table S1. Accession numbers for data downloaded from the European Nucleotide Archive. Human data are from the 1000 genomes project [36]. Taxa for which transcriptome data was used are italicized. [file 12859_2015_632_MOESM3_ESM.pdf]

Species	Data from the 1000 Genomes Project		
**Human**	20120117_ceu_trio_b37_decoy: NA12878*	pilot2_high_cov_GRCh37_bams: NA19238*	
	Accession Numbers		
**Bonobo**	ERR018599	ERR032964	
**Chimp**	SRR490082	SRR490117	
**Crab Macaque**	SRR445602	SRR445694	
**Gorilla**	ERR225700	ERR223709	
**Orangutan**	SRR032888	ERR247212	
**Rhesus Macaque**	ERR247210	SRR392234	
**Aardvark**	SRR360815	SRR360820	
Armadillo	SRR494766	SRR494775	SRR309130
**Mysticeti (Baleen whales)**	SRR893003	SRR935201	
Microchiroptera (microbats)	SRR539297	SRR617081	
Felidae	SRR924676	SRR586041	
**Colugo**	SRR585693	SRR593622	
Cow	ERR315616	SRR361433	
Dog	DRR001151	SRR094922	
Elephant	SRR027944		
**Elephan shrew**	SRR387354	SRR387355	
Horse	SRR495385	SRR495394	
Human	ERR315325	ERR315344	
Lemur	SRR361350	SRR361352	
Macaque	ERR247244	SRR223515	
**Manatee**	SRR331132	SRR331147	
**Megachiroptera (megabats)**	SRR534483	SRR534546	SRR924356
Mouse	SRR361355	SRR361357	
Opossum	ERR039158	ERR039159	
**Pangolin**	SRR770301	SRR770333	
Pig	SRR543893	ERR208926	
Pika	SRR850199	SRR850200	SRR850201
Rabbit	ERR162219	SRR388298	
Rat	SRR850240	SRR850609	
Shrew	SRR765874	SRR765877	SRR869610
Sloth	SRR857575	SRR857865	
Star-nosed mole	SRR353136	SRR392033	
Tenrec	SRR107098	SRR107099	
Odontoceti (toothed whales)	SRR653403	SRR653420	SRR027945
Treeshrew	SRR363081	SRR363088	
Wallaby	DRR013408	DRR013424	
